# Hysteria and Brain Tumors1Read at the thirtieth annual meeting of the Medical Association of Central New York, held at Buffalo, October 19, 1897.

**Published:** 1898-08

**Authors:** Wm. C. Krauss

**Affiliations:** Buffalo, N. Y. 371 Delaware Avenue


### HYSTERIA AND BRAIN TUMORS.[1]

BY WM. C. KRAUSS, M. D., Buffalo, N. Y.

WHERE the classical symptoms of brain tumor, as optic neuritis, headache, vomiting, hebetude and vertigo are present, it is perhaps inexcusable for the physician to mistake the process in the brain ; but where one or two of these characteristic symptoms are absent or illy-developed, speculation as to the diagnosis is not only permissible but commendable. It is only in certain rare cases where the question of differential diagnosis between organic brain disease (tumor) and functional nervous disease (hysteria) arises that great care and skill must be exercised in the plan and in the grouping together of the results of the examination. Neither is it advisable nor discreet to commit one's self after a single cursory examination, such as is generally offered at consultations, but several thorough examinations should be insisted upon before the diagnosis is finally made. To call a case of tumor,—functional,—and promise an almost certain recovery, and then in the course of a few weeks be invited to attend the necropsy is certainly jarring to one's sensibilities ; on the other hand, to discover the exact location of a tumor, advise operation and then watch the patient rapidly recover in the few days preceding the operation, or to find a healthy brain cortex in the trephine opening and the tumor symptoms all vanish after the cicatrisation of the wound is no doubt a great medical or rather medico-chirurgical triumph, but one in which the neurologist or attending physician plays no part. It might seem preposterous for a brain lesion, meaning certain death, to be mistaken for a functional nervous disease, generally curable and vice versa, and yet those who have had much experience in brain diseases will admit that such mistakes are liable to occur.

Schönthal[2] reports the case of a young man of nervous tendencies who began to fall into convulsions of hysterical character after a

1. Read at the thirtieth annual meeting of the Medical Association of Central New York, held at Buffalo, October 19, 1897.

2. Berliner Klinische Wochenschrift, 1891, No 10.

domestic quarrel. These spells were general, affecting the whole body, sometimes assuming opisthotonus. There was no history of head pain, vomiting, optic neuritis or mental torpor. The patient was of a susceptible temperament and was accordingly treated as a case of ordinary hysteria. After an illness of several months the patient died of an hypostatic pneumonia. At the autopsy, besides the expected pulmonary lesion an unexpected tumor was found in the left frontal lobe, which proved to be a hemorrhagic glioma.

Another interesting case was presented by Thomas[1] before the Johns Hopkins Hospital Medical Society, May 17, 1897, from which I quote in extenso :

The patient whom I wish to present to the society is a young, unmarried woman of 26, with a good family history, except that one brother is believed to have tuberculosis. She has been a fairly strong girl and has had no serious illness of any kind. Her occupation is that of a sewing woman and she has been learning stenography and typewriting.

About a year ago she had an attack of unconsciousness; this began with a painful contraction of the right hand, which lasted for a minute or two and passed up the arm, when she became unconscious. She was found about half an hour afterward by her brother, who shook her and brought her to herself. After this attack she was comparatively well and went to her employment the next day as usual. In August of last year she was under a great deal of nervous strain on account of the death of a relative. About this time she had several nervous attacks, which she calls "nervous chills," and which seem to have been hysterical. On November 8, 1896, while sitting with her arm resting on the table, she became conscious of a sensation of numbness in the elbow of her right arm. This arm and the leg on the same side became suddenly powerless and she has been told that her face was drawn to the left, but of this she was not conscious. With the onset of this paralysis she became absolutely unable to speak. She was put to bed and showed so much nervousness that the attending physician, a very competent man, made the diagnosis of hysteria and it was impressed upon her that she could, if she would, get up and walk and talk.

The 1st of last December she had another attack and another on the 12th of February and still another on the 26th of April. These attacks have all been of the same character, beginning with a painful contraction of the fingers of the right hand; this passes up the arm and she describes an intense pain in her head. At this time she says that she loses all knowledge that the arm belongs to herself, although she still experiences pain in it.

The ophthalmoscopic examination showed that the fundus was normal, although there is a slight degree of hyperemia. She has a congenital squint and a slight nystagmus, but other than this, no abnormality of the cranial nerves. She is unable to oppose the thumb to the little finger and the movements of the finger are very weak. The movements at the wrist are better retained and those at the elbow and shoulder better still. The movement of outward rotation of the arm is very weak indeed, whereas the inward rotation is comparatively strong. The deep

1, John Hopkins Hospital Bulletin, October, 1897.

reflexes are markedly exaggerated, percussion on any of the tendons causing active muscular contraction, and there is a well-marked wrist clonus. Objectively, there is not much muscular weakness of the right leg. Sensation is everywhere perfectly normal.

The author sums up the case as follows, showing that a large element of doubt has crept into the case, although he believes he has to deal with a "*probable* brain tumor." "I have been much interested in this patient, and it is surely an unusual case. That a young woman of her age should have been affected with a hemiplegia associated with a temporary loss of speech, and followed by recurring attacks, simulating closely Jacksonian epilepsy, is very remarkable. The diagnosis ot hysteria seems at first sight the most probable one. Organic vascular lesions are veiy rare at her age, except when they are associated with syphilis. In this case there is nothing in the previous history or in the present condition that suggests such a cause. The patient was at the onset undoubtedly very hysterical, and you will remember that she gave the history of preceding hysterical attacks, all of which speak for the diagnosis of hysteria, but the condition in which you have seen the patient suggests an organic paralysis more than an hysterical one. Mann and other observers have studied the distribution of the paralysis, due to organic brain disease, and have determined that there are certain movements which are most apt to be paralysed. The movements which are most often paralysed are those of the thumb, and next in order of importance is the outward rotation of the arm. In the case before you, these are just the muscles which are paralysed. The excessive increase of the deep reflexes, which is present in this case, is also unusual in association with hysterical paralysis, so I think we are justified in stating that the character of the paralysis is that due to an organic brain lesion rather than to hysteria. The character of the convulsive attacks also points to an organic lesion, as true Jacksonian attacks are, as far as my knowledge goes, extremely rare in hysteria. Although *I am not very positive about it*, still I believe that we have a definite disease of the brain in this case, and I am inclined to think that this disease is a slow growing tumor."

Another case[1] reported from Dr. Ball's clinic in Saint Luke's Hospital, New York:

The patient was a girl, 16 years of age, who had suffered from vomiting and headache for about ten weeks before her death. During the last four weeks of her life she was under treatment and thought to be suffering from chronic gastritis

1. Medical and Surgical Report of the Presbyterian Hospital, New York, Vol. I., January, 1896, p. 78.

and hysteria. The headache was severe and persistent. The vomiting was most frequently in the morning, occurred from one to many times in the day, was independent of the taking of food or other apparent cause, and was generally accompanied by nausea. She was irritable and apparently hysterical. A week before her death she appeared to be much improved. About seven hours before her death she complained of severe pain in the head, the back of the neck and the epigastrium and was very irritable and noisy. She then became unconscious, with a general rigidity of the body, which lasted an hour; the pupils became much dilated, more so on the right side; there were slight muscular twitchings and for fifteen minutes a general tonic convulsion; the respiration became labored and the surface cyanosed.

At the autopsy a brain tumor was discovered. Along the upper inner edge of the right temporal lobe was a new growth, measuring 2 x 1 x 1 inches. It formed a part of the floor of the descending horn of the lateral ventricle and invaded the brain tissue in the neighborhood. About the growth, the tissues were degenerated and cystic. The right crus cerebri was compressed, atrophied and softened; the posterior part of the right optic thalamus was pressed upward and forward and the corpora quadrigemina were pushed toward the left and flattened; the convolutions of the cerebral hemispheres were much flattened. Microscopical examination discovered the structure to be a papillary growth, the vascular projections of connective tissue being covered by a layer of regular cylindrical cells.

This case resembles somewhat a case of my own where the diagnosis was at first thought to be that of hysteria, where improvement apparently followed treatment and where at the autopsy the brain was found to be the seat of advanced and extensive pathological change.

Thoma[1] has reported three cases of psychoses developing in individuals between the fiftieth and fifty-eighth year, where a decided hysterical element pervaded the symptoms without any suggestion as to any brain complications, and yet at the autopsy a brain tumor was found in each case.

Within a short time I have seen several cases in consultation which will illustrate some of the difficulties encountered in these illy-developed brain cases, resembling functional trouble and in functional disturbances simulating brain disease. One of the most pronounced is the following case:

Miss L., age 21; height, five feet one inch; weight, 120 pounds; complexion, dark; constitution rather delicate. Family history: grandparents lived to old age; parents living and healthy; no history of cancer, syphilis or tuberculosis obtainable. Early history: she passed through the diseases of childhood without any sequelæ; menstruated at thirteen years. She was bright in her school work and was employed for several years as a teacher. A love affair, with disappointing consequences, prompted her to cease her work and she left her home, near Canisteo, N. Y., for Rochester, N. Y., to resume her studies, in the fall of 1895

1. Neurologisches Centralblatt, 1896, p. 38.

While here she began to complain of not feeling well, was nervous, irritable, cross, suffered from headaches a great deal of the time and was losing in flesh. Her physician, diagnosticating her condition as neurasthenia with hysteria, prescribed nerve tonics and advised her to return to her home in the country. For a short time the change from city to country seemed to do her good and she became less nervous and complained less frequently of the head. In the spring of 1896 she began, however, to grow worse, the headaches, limited to the right side of the head, became more intense and at times she would have vomiting spells. She would also have attacks of syncope, especially if irritated or tired by callers. The left arm and leg became numb and she found that she was losing power over them. The fainting spells and head pains of the right side becoming so frequent and so intractable, a council of physicians residing in the neighborhood was called and after considering her case carefully decided it was hysteria. The head pains were regarded as hemicrania and the paresis of the left arm and leg was looked upon as of functional character. The fainting spells were supposed to be hysterical convulsions. They prescribed antispasmodics—dashing the face with cold water while in one of the fainting spells—and ordered absolute rest and seclusion.

This information was tendered me by the attending physician. Not satisfied with the condition of the patient, who seemingly was not improving, I was called by the brother to go and make a careful examination of the patient and report my conclusions to him.

On May 10, 1896, in the presence of the attending physician, I made the following examination: The patient, a bright, cheerful looking young lady, was in bed with cold compresses, soaked in alcohol, pressed against the right side of the head, while the left arm lay motionless upon the bed spread. She seemed to be in the best of spirits, laughed, joked and evidently anticipated having a good time. Her countenance was cheerful, eyes were sparkling and she seemed to enjoy her surroundings and home. Her mind was clear, active and alert; memory was good and her conversation interesting and entertaining. Head was very sensitive to touch—even the hair—and any pressure over the right side of the head was unbearable. In short, a degree of hyperesthesia was present. Eyes: pupils were equally dilated and no ocular defect of any kind could be noted. Unfortunately, not knowing the nature of the case, I did not bring an ophthalmoscope along and as the fundus had never been examined, it was impossible to tell in what condition the optic discs were. She did not complain of any loss of vision—thought she was able to see as well as before her sickness. The lower part of the face, left side, appeared somewhat paretic and the tongue deviated some to the left. The left arm was powerless, but when urged to exert herself she could move it and could develop some grip in the left hand. She could draw the left leg up, but could not raise it from the bed. The muscles were well developed and of good consistency. Tendon reflexes were slightly increased on both sides. There existed some difference in the sensation between the right and left sides, but not sufficient to attract much attention. Bowels were constipated; appetite poor; sleep irregular; urine normal; also pulse and temperature.

The examination was extremely unsatisfactory, because the one fact which I wished to know was unobtainable—namely, the condition of the optic discs. I informed the attending physician that the patient had many symptoms that might be regarded as hysterical, that the supposed cause (a love affair) the hyperesthesia, the fainting spells, the appearance and demeanor, the paralysis of the arm and leg even, all could and are frequently met with in young girls of hysterical tendencies. On the other hand, there were indications that a more serious condition was present, and that a tumor in the right motor areas was probable. The latter view was the stronger and yet I did not dare inform the parents of the probable tumor and consequent prognosis unless I was reasonably sure. The ophthalmoscope only could decide the question in my estimation and as the patient's condition did not seem dangerous it was decided to have another examination on the following Sunday. The trip was postponed for another week, but on May 21st I received word that the patient died in one of the fainting spells. An autopsy was not allowed, but there is no doubt that the fatal termination decided the question unconditionally.

In some respects the case resembles another case of my own where the diagnosis was at first thought to be some functional disturbance but later judged organic by the appearance of the optic neuritis. The autopsy confirmed the opinion, as several tumors were found scattered throughout the brain. This case also is noteworthy because of the patient's mental condition. Instead of finding dulness of intellect, apathy, heavy countenance, dull expressionless eyes, the patient was an active, bright and sprightly girl with dash and fire in her eyes and possessed of a mind without any care. The fainting spells also deserve attention and is an unusual symptom in cases of brain tumor. It is needless to speculate upon the exact location of the tumor or its character as both facts must always remain problematical.

All cases of suspected brain tumor with hysterical manifestations must not be considered as having been absolutely organic because death has occurred, since it is a well-known fact that a fatal termination may sometimes result from the different effects of hysteria, and it is quite a mistake to look upon the disease as always having a favorable prognosis, so far as life is concerned.

Fournier and Sollier have observed cases of spasm of the glottis in hysterical girls so severe that death ensued. Also in hysterical angina pectoris, which is generally curable, yet Potain reported a case in which death took place and in which on post mortem examination absolutely nothing was found.

Fournier and Sollier also refer to hysterical anorexia in which there is sometimes a fatal termination, and even should recourse be had to artificial feeding there seems to be no power of absorption. The wasting continues and the patient dies. They also refer to the danger of forcible feeding in such cases, and one of their patients who presented a marked degree of anorexia expressed a wish for some cheese, and died the same evening that she ate it. The authors point out that sudden death may occur after hysterical vomiting, and they give the notes of one of such cases, no lesion of any kind being found on post mortem examination. Thus it will be seen that the utmost care is necessary in making the examination and still greater care and caution in interpreting the meaning of the different symptoms. In a previous paper I have called attention to three groups of symptoms occurring in tumors of the brain, viz.: the early symptoms, the classical symptoms and the decisive symptom.

The early symptoms are similar to those met with in neurasthenia and hysteria, as headaches, incapacity for mental work, disordered digestion, nervous irritability and a general malaise. The classical symptoms enumerated in the order of their importance are (1) head pain, (2) optic neuritis, (3) mental apathy, (4) nausea and vomiting and as a special localising symptom to be added to this group must be included (5) paralysis. The decisive symptom, choked disc, is the only symptom which has never been observed in the varied symptomatology of hysteria, whereas all the early and classical symptoms have been frequently noted in functional diseases. It is therefore of extreme importance that this sign should be sought for, not only at the first examination, but at every subsequent examination until its presence is determined, or its absence along with continued improvement signifies a purely functional disturbance in the patient. It is well illustrated in the following interesting case: I was recently called into consultation to see a middle-aged lady who was suspected of having some cerebral neoplasm and was directed to localise the growth so that an operation could be performed. The patient had complained for some months with a severe headache over the right parietal bone, sensitive on pressure, with spells of nausea and vomiting. Her mind was dulled, her voice almost inaudible, and it was with some difficulty that she could be persuaded to carry out the orders of the physician. There was no pronounced paralysis of the extremities present, although the tongue seemed to point more toward the left and the velum palati over the right side seemed higher than on the left. The pupils were equally dilated, reacted normally and the ophthalmoscope failed to show the least disturbance of the optic discs. No satisfactory results could be obtained from the cutaneous examination on account of the apathy and blunted mentality of the patient.

The diagnosis of hysteria was made almost exclusively on the absence of papillar disease and subsequent improvement and recovery substantiated the correctness of the diagnosis.

Studying carefully 100 cases of brain tumor in which an ophthalmoscopic examination had been made for the presence or absence of choked disc (optic neuritis) I was forced to the following conclusions:[1] (1) Optic neuritis is present in about 90 per cent. of all cases of brain tumor. (2) It is more often present in cerebral than in cerebellar cases. (3) The location of the tumor exerts little influence over the appearance of the papillitis. (4) The size and nature of the tumor exerts but little influence over the production of the papillitis. (5) Tumors of slow growth are less inclined to be accompanied with optic neuritis than those of rapid growth. (6) It is probable that unilateral choked discs is indicative of disease in the hemisphere corresponding to the eye involved. (7) It is doubtful whether increased intracranial pressure is solely and alone responsible for the production of an optic neuritis in cases of brain tumor. (8) Optic neuritis is never present in functional nervous disease. (9) Where the diagnosis between organic brain disease (as tumor) and functional nervous disease (as hysteria) is held in abeyance, the presence or absence of optic neuritis in the great majority of cases will clear up the doubt.

371 DELAWARE AVE.

